# Awareness on breast cancer screening in Malaysia: a cross sectional study

**DOI:** 10.1051/bmdcn/2019090318

**Published:** 2019-08-27

**Authors:** Mun-Seng Lee, Choiriyatul ‘Azmiyaty Amar Ma’ ruf, Dayang Puteri Nadhirah Izhar, Sayyida Nafisah Ishak, Wan Syazana Wan Jamaluddin, Syafiqah Nadiah Mohd Ya’acob, Muhammad Nazrullah Kamaluddin

**Affiliations:** 1 Quest International University Perak Ipoh Perak Malaysia

**Keywords:** Breast Neoplasms, Mass screening, Awareness, Knowledge and Attitude

## Abstract

Introduction: The increasing rate of breast cancer (BC) incidence in Malaysia hints a lack of awareness among Malaysians. One (1) woman out of nineteen (19) is at risk with BC and almost up to fifty percent (50%) of women diagnosed with BC were reported to be under the age of fifty (50). Our main concern is to study the level of awareness among the women on risk factors, clinical manifestations, diagnosis, preventions and treatments.

Method: A cross-sectional study was conducted exclusively among women in the public with total sample of three hundred and forty six (346), questionnaires were distributed using a simple random technique. Data was collected and analyzed by student *T* test in SPPS version 20.

Results: Our study reveals insufficient awareness on BC. Overall, awareness on risk factors is inadequate, but good knowledge on the importance of family history and diet as risk factors are discovered. Awareness on the cause and clinical manifestations of BC is required for improvement. As for treatment, alternatives especially surgery and chemotherapy are unclear to public, public is remotely unwitting on cessation of smoking to prevent BC at the early stage.

Conclusion: Malaysian has spaces for improvement on awareness of BC in terms of risk factors, clinical manifestations, diagnosis, treatment and prevention. Early detection can be achieved with good awareness because it leads to better prognosis and lower mortality.

**Abbreviations**
ACS: American Cancer Society
BC: Breast Cancer
BSE: Breast Self-Examination
CBE: Clinical Breast Examination
CDC: Centre for Disease Control and Prevention
CPG: Clinical Practice Guideline
CRUK: Cancer Research UK
DNA: Deoxyribonucleic Acid
HRT: Hormonal Replacement Therapy
MOH: Ministry of Health
OCP: Oral Contraceptive Pill
PhD: Doctor of Philosophy
PM: Penn Medicine
PMR: Penilaian Menengah Rendah
SPM: Sijil Pelajaran Malaysia
SPSS: Statistical Package for the Social Science
WCRF: World Cancer Research Fund
WHO: World Health Organization

## 1. Introduction

Malaysia has a high prevalence of breast cancer (BC), one (1) in nineteen (19) women is at risk with BC, as it is the most commonly diagnosed cancer among women of all ethnic groups [1, 2]. According to the World Health Organization [3], from the year 2002 to 2012, the number of BC cases was recorded the highest (five thousand four hundred and ten cases) among Malaysian women as compared to other cancer cases involving the cervix uteri (two thousand one hundred and forty five cases), colorectal (one thousand nine hundreds and seventy six cases), lung (one thousand one hundred and sixty three cases) and ovary (one thousand and ninety eight cases). It showed that the age-standardized death rates per one hundred thousand (100,000) due to BC was the highest consecutively throughout the decade reaching the peak rate of twenty-three percent (23%) between the year 2004 and year 2006. In Malaysia, up to fifty percent (50%) of women were diagnosed with BC were reported to be under the age of fifty (50) [1, 4–6].

Three (3) breast screening methods are recommended, these include clinical breast examination (CBE), mammography for women over forty (40) year-old and breast self-examination (BSE) [7–10]. Developing countries are known to promote BSE as an essential approach for early detection of BC but the mammography screening is unavailable due to limited resources [11–14].

Our main concern is to study the level of awareness among public in terms of knowledge of risk factors, clinical manifestations and self-examination for early detection. Different education level affects a person’s perspective on BC differently, it is undeniably important to identify general awareness in the public especially the women and emphasized on early detection.

## 2. Objective

Our study is driven by three important objectives. Firstly, evaluation on the level of BC awareness among Malaysian women is crucial. Secondly, further discussion on every aspect of BC in Malaysia are carried, these include risk factor, cause, clinical manifestation, diagnosis, treatment and prevention. Lastly, assessment on the current situation of BC in Malaysia as a whole is pursued.

## 3. Method

A cross-sectional study was conducted among women in the public area. We managed to collect 346 samples. Our exclusion criteria were male and those female less than 21 year-old because anyone below 21 year-old was still consider as minor in Malaysia. Data was collected by a simple random technique to pursue a cross sectional study. A set of questionnaire was modified from “Breast Module of the Cancer Awareness Measure (Breast - CAM) Toolkit” [15] to screen awareness of BC among Malaysian women. The questionnaires have been delivered bilingually, which are in English and Malays and analyzed by Statistical Package for the Social Science (SPSS) version 20. The participants have given their consent by filling a form with guaranteed confidentiality. Each questionnaire consisted of thirty-eight (38) questions along with other demographic background information from each participant. Each answer was rated by Likert’s scale from 1 to 5 whereby 1 indicated “strongly disagree” while 5 expressed as “strongly agree”. Instructions were given to the participants to score each question based on their knowledge from 1 to 5. Student *T* test was deployed for each section, the overall Cronbach’s alpha reliability was analysed to justify our study.

## 4. Results

According to Table 1 55% of the respondents consist of women age ranging from 21 to 30 year-old, 15% are women at the age of 31 to 40, 11.6% are of the range 41 to 50 year-old, followed by 11.3% in the range of 51 to 60 year-old, and the least respondents are of the age of 61 to 70 year-old which is 7.2%.

**Table 1:** The demographic profile of the respondents.

Demography	Category	Percentage (%)
Age	21-30	54.9
	31-40	15.0
	41-50	11.6
	51-60	11.3
	61-70	7.2

Race		62.7
	Malays	17.9
	Chinese Indian Others	13.6
		5.8

Marital status	Single	56.9
	Married	43.1

Education level	No formal education	0.9
	PMR	4.6
	SPM	24.3
	Pre-University	26.6
	Undergraduate	37.0
	Master	5.5
	PhD	1.2

Employment	Employed	52.6
	Unemployed	47.4

Past History of BC	Yes	1.4
	No	98.6

PMR: Penilaian Menengah Rendah; SPM: Sijil Pelajaran Malaysia; PhD: Doctor of Philosophy.

Regarding ethnicity, the highest respondents are recorded among Malays which covers 62.7% of the respondents, followed by Chinese, 17.9% respondents. Meanwhile, 13.6% respondents are Indians and 5.8% are from other races. 56.9% of respondents are found to be single and 43.1% married.

In terms of education, 0.9% respondents have never acquired formal education, 4.6% studied up to Penilaian Menengah Rendah (PMR) level, 24.3% studied up to Sijil Pelajaran Malaysia (SPM) level, 26.6% are still in their pre-university studies, 37.0% are undergraduates, 5.5% respondents are postgraduates and 1.2% are Doctor of Philosophy (PhD) holders. SPM is equivalent to high school final year examination, PMR is equivalent to high school third year examination. In addition, 52.6% of the respondents are employed, the rest 47.4% are unemployed.

Most of the respondents never have a history of BC in their lifetime, they account for 98.6%, only 1.4% respondent experienced BC.

Our Cronbach’s alpha reliability test result is 90.8%, this suggests a good reliability of our study. Our study is designated with 95% confidence interval and the margin error of 6%.

This section is to assess the awareness of Malaysian women on risk factors. In Table 2 it points less aware on women age between forty (40) to sixty (60) year-old at risk of BC with the mean score of 3.5376, it is statistically significant (p < 0.05). Female gender is a recognised risk for BC, women are well-informed of this fact with score mean above 4 but it is not statistically significant *(p* > 0.05), public may not have a good understanding on this risk.

**Table 2:** Risk factors of BC.

Risk factors	Mean	Std. Deviation	Std. Error Mean	Sig. (2- tailed)
Age 40-60	3.5376	0.92001	0.04946	0.000
Female gender	4.0173	0.92297	0.04962	0.727
Benign breast disease	3.3815	1.00382	0.05397	0.000
Diet- alcohol, saturated fats, smoked meat	3.9306	0.88847	0.04776	0.147
Early menarche	2.7803	0.98291	0.05284	0.000
Late menopause	2.7746	0.93625	0.05033	0.000
Late childbirth (after 30 years/ nulliparity)	2.8121	1.01123	0.05436	0.000
Hormone replacement therapy (HRT)	3.2659	0.95331	0.05125	0.000
Absent breastfeeding	3.1994	1.09708	0.05898	0.000
Oral Contraceptive Pills (OCP)	3.1908	0.96521	0.05189	0.000
Family history of BC	3.9566	0.94232	0.05066	0.393
Carcinoma of endometrium or uterus	3.3295	0.97273	0.05229	0.000
Post radiation exposure	3.6329	0.93620	0.05033	0.000
Sedentary life style (lack of exercise)	3.5607	0.90285	0.04854	0.000

Oestrogen and non oestrogen related risk factors are not publicly familiarised as shown in Table 2. Oestrogen related risk factors include benign breast disease, early menarche, late menopause, late childbirth showed, desisted breastfeeding and use of HRT or OCP. Whereas, non-estrogen related risk factors are comprised of the history of carcinoma of endometrium, post radiation exposure and sedentary lifestyle. Our finding is statistically significant *(p* < 0.05), these risk factors are publicly unwitting.

As regarding to diet factor and family history of BC, both of them has a mean score of 3.9306 and 3.9566 respectively. However, they are not statistically significant (p > 0.05), this suggests that women are unaware of diet and family history of BC to be risk factors.

Table 3 indicates women in Malaysia are not aware on cause of BC such as damage in the BC deoxyribonucleic acid, our result is statistically significant. It also shows the results of assessing respondent’s knowledge on clinical manifestations of BC. Although the mean score for armpit lump and nipple discharge or bleeding denotes lack of awareness, it is not statistically significant (p > 0.05). This insinuates that the respondents actually have some degree of awareness on these symptoms of BC. As regarding to breast lump symptom, the respondents express a good awareness, it is statistically significant *(p* < 0.05). Unfortunately, the respondents are unwitting on the BC non-lump symptoms such as nipple position changes, nipple retraction, puckering or dimpling of the breast, breast pain, armpit pain, nipple rash, lump under armpit, redness of breast skin, change in size and shape of breast and nipple because it is statistically significant *(p* < 0.05).

**Table 3:** Cause and Clinical Manifestations of BC.

Cause	Mean	Std. Deviation	Std. Error mean	Sig (*p* value)

Damage of breast cell Deoxyribonucleic Acid (DNA)	3.5607	0.85673	0.04606	0.000

**Clinical Manifestations of BC**	**Mean**	**Std. Deviation**	**Std. Error Mean**	**Sig. (*p* value)**

Change in nipple position	3.5809	0.93597	0.05032	0.000
Retraction of nipple	3.4740	0.97545	0.05244	0.000
Breast or armpit pain	3.8699	0.83605	0.04495	0.004
Puckering or dumpling of breast	3.7197	0.84409	0.04538	0.000
Discharge or bleeding from nipple	3.9451	0.79118	0.04253	0.198
Breast lump	4.0867	0.77534	0.04168	0.038
Nipple rash	3.5087	0.85884	0.04617	0.000
Redness of breast skin	3.5925	0.84706	0.04554	0.000
Lump under armpit	3.9162	0.77753	0.04180	0.046
Change in size of breast or nipple	3.6705	0.85540	0.04599	0.000
Change in shape of breast or nipple	3.8237	0.83448	0.04486	0.000

Diagnosis is an important aspect in medicine in order to identify and confirm any morbid conditions. A few ways to diagnose a patient include a standard regular CBE, routine mammography and laboratory tissue. In Table 4, the mean scores for CBE and routine mammography demonstrate an excellent level of awareness on the knowledge of diagnosing BC. As regarding to tissue biopsy, the mean score shows that they are not aware of this practice in diagnosing BC (p > 0.05). This is a negative public perception but the community might have a possible good awareness regarding this procedure.

**Table 4:** Diagnosis of BC.

Diagnosis	Mean	Std. Deviation	Std. Error Mean	Sig. (*p* value)
CBE	4.2139	0.73853	0.03970	0.000
Routine Mammography	4.1879	0.80719	0.04340	0.000
Tissue Biopsy	3.9769	0.90059	0.04842	0.633

Various types of treatment are available for BC patients. In Table 5, the treatments include chemotherapy, hormone therapy, radiation therapy and surgery. Patient may have awareness on chemotherapy but there is a possibility of misunderstanding on the treatment itself (p < 0.05). Meanwhile, the mean scores of hormone therapy and radiation therapy represent low awareness among the women in Malaysia on the knowledge of treatments in BC (*p* > 0.05), it can be due to false perception on this particular treatment basis of BC, they may have a good understanding on this aspect. Similarly in radiotherapy and hormone therapy (*p* > 0.05), they may have minimal knowledge on these aspects. The mean score for surgery signifies that these women are aware on this era (p > 0.05), public may have a misinterpretation on surgery.

**Table 5:** Treatment of BC.

Treatment	Mean	Std. Deviation	Std. Error Mean	Sig. (*p* value)
Chemotherapy	4.0318	0.91470	0.04917	0.518
Hormone therapy	3.6012	0.89921	0.04834	0.000
Radiation therapy	3.7861	0.86170	0.04633	0.000
Surgery	4.0780	0.78923	0.04243	0.067

Table 6 lists down the respondents view on the preventive measures on BC. The mean score of smoking cessation denotes good awareness (*p* > 0.05), it signifies that public generally may not have a good understanding on cessation of smoking to prevent BC. The mean scores of all other prevention methods such as regular BSE, mammogram, practising a healthy diet and regular exercise symbolise that public has a good awareness on the prevention of BC (p < 0.05).

**Table 6:** Prevention of BC.

Prevention	Mean	Std. Deviation	Std. Error Mean	Sig. (*p* value)
Regular BSE	4.3208	0.82249	0.04422	0.000
Mammogram	4.1705	0.78563	0.04224	0.000
Halt smoking	4.1040	0.92353	0.04965	0.037
Healthy diet	4.2977	0.83811	0.04506	0.000
Regular exercise	4.1908	0.85365	0.04589	0.000

## 5. Discussion

### 5.1. Demography

In our study, elder people are preoccupied with higher expectations in health studies, they prefer a study to be well-prepared and polished. They expect interviewers to perform their task in a professional, polite, positive and knowledgeable manner. They are not keen to pursue if an interviewer is unconfident, timid and approaching weakly. It is essential to train interviewers to be sensitive to these matters, this improves their ability of building a better rapport with the respondents [16–19]. Our data is aligned with the country’s population distribution. More than half of the respondents are single, our participants are of the younger age groups. There is minimal gap between the employed and unemployed, some of the unemployed respondents are pursuing their studies.

### 5.2. Risk factors

There are fourteen (14) risk factors of BC put up in our questionnaire. Based on our result, public is only aware of two risk factors out of the fourteen (14) such as family history of BC and diet factor. According to our results, women are slightly incognizant that female gender is a risk factor for BC. Our study shows a good awareness on dietary factor. High alcohol consumption has a very strong association towards developing BC because drinking leads to enhanced permeability of membranes to carcinogens thus escaping from detoxification [20–24].

Unfortunately, public awareness on radiation therapy is less ideal. Women with younger age and treated with radiation therapy to the chest for previous or another cancer have a significantly higher risk for BC [25–27], but radiation treatment after forty (40) year-old does not increase BC risk [28]. A sedentary lifestyle with lack of physical activity and practising an unhealthy diet potentially lead to BC, but public is unwitting on this particular fact in our study. Women with inherited mutation in PTEN gene, which causes Cowden syndrome, are at high risk for both non- caner and cancer tumours in the breasts, in the digestive tracts, thyroid, uterus and ovaries [28]. In our study, public is insensitive to endometrial cancer or cancer of the uterus being a risk factor of BC.

In our study, public is unperceptive on potential risk factors of BC. Misconception is our social dilemma, our society regards cancer to be scary and negative. It is a taboo in the culture and society whereby people prefer to keep things silent in spite of strong positive family history [29, 30].

### 5.3. Cause

In our study, public have insufficient awareness on damage of breast cell DNAleading to BC. Educational level and socioeconomic status play important role on awareness, cancer awareness is suboptimal among those who are less well educated and those with a lower socioeconomic status [31, 32].

### 5.4. Clinical manifestation

Three factors influence women’s interpretations on the clinical manifestation of BC, they include symptom nature, knowledge of women and perceptions of women on getting BC. If the symptom is incongruent with these factors, this lead to action thus preventing delay in seeking medical help. Women usually regard the clinical manifestation of BC as a non-serious condition or an ordinary ailment if they do not have knowledge on them, these factors should be fulfilled and emphasised among women [33, 34]. Unfortunately in our study public is not aware of non-lump symptoms as the clinical manifestation of BC.

### 5.5. Diagnosis

Our study reveals a good public understanding on the screening methods for BC especially CBE and routine Mammography but they have a negative perception towards biopsy. They believe this procedure is an invasive procedure, it is stigmatized to painful in spite of good benefits. Mammography is presumed to be painful by peers, healthcare professionals are vital to deliver adequate information on this screening method in order to eliminate this particular stigma [35, 36].

### 5.6. Treatment

In our study, BC treatments are inadequately perceived. Public may have false conception or lack of exposure on treatments, they should be informed in depth. Public may have low interest to explore, they prefer to familiarize themselves with complementary and alternative medicine [37, 38]. Apart from surgery, public is hesitated on chemotherapy for BC. Public voices out their fear of cancer consequences, the pain from chemotherapy and surgery are perceived as a death punishment [39, 40]. These negative perceptions prevent them to pursue chemotherapy and surgery as treatments of BC. Public is also unmindful on radiotherapy and hormone therapy due to insufficient and incomplete knowledge. Promotions on breast health awareness should be advocated thoroughly in our country, so that the knowledge about treatment of BC is accessible to public in both rural and urban areas [41].

### 5.7. Prevention

As according to our result, public is well-versed with the strategies of preventing BC. Our study intimates a good public awareness on mammogram screening to prevent BC. In our study, public has a good awareness to practise a healthy diet. Regular exercise prevents BC, mammary carcinogenesis can be inhibited by caloric restrictions *via* physical activity since it affects energy balance [42, 43]. Public is well aware of regular exercise is one of the preventive measures for BC in our study.

Public is unwitting on cessation of smoking to be a preventive measure for BC. Unfortunately, tobacco companies elaborate their health message on the correlation between lung cancer with smoking instead of BC, this misleads public to compartmentalize tobacco-related risk to a specific area of the body [44, 45].

## 6. Conclusion

In summary, public still have spaces for improvement on awareness of BC. They may need further knowledge, but promotion in early detection is essential because there are many risk factors and clinical presentations. Social taboo among women themselves on discussing matters of BC or unsuccessful promotion of BC by healthcare providers may be possible obstacles. Women may be reluctant or choose to be ignorant on knowing BC in depth albeit.

There are many massive BC awareness campaigns in the country, they should focus on the dangers on ignoring BC instead of promoting the knowledge of presentation in BC. Both women and health care providers should work hand in hand to combat against the mortality and morbidity of BC.

## Conflicts of interest statement

The authors wish to disclose no conflicts of interest.

## References

[1] Lim GC , Second Halimah Y . Report of the National Cancer Registry. Cancer Incidence in Malaysia. 2003: 1–41.

[2] Howlader N , Noone AM , Krapcho M , Miller D , Bishop K , Kosary CL , et al SEER Cancer Statistics Review, 1975–2010. Bethesda, MD: National Cancer Institute 2013: 21, 12.

[3] Population Reference Bureau. 2016 World Population Data Sheet; Washington, DC; PRB, 2016.

[4] Gco.iarc.fr. (2019). Malaysia Fact Sheets. [online] Available at: http://gco.iarc.fr/today/data/factsheets/populations/458-malaysia- fact-sheets.pdf [Accessed 20 Mar 2019].

[5] Hisham AN , Yip CH . Spectrum of Breast Cancer in Malaysian Women: Overview. World J Surg. 2003; 27(8): 921–3. 12784146 10.1007/s00268-003-6976-x

[6] Looi LM , Zubaidah Z , Cheah PL , Cheong SK , Gudum HR , Iekhsan O , et al Research on Cancer Diagnosis in Malaysia: Current status. Malays J Pathol. 2004 Jun; 26(1): 13–27. 16190103

[7] Narimah A , Rugayah HB , Tahir A , Maimunah AH . Breast Examination, National Health and Morbidity Survey 1996, Volume 20; Malaysia; Kuala Lumpur; Ministry of Health; Public Health Institute, 1999.

[8] Takkar N , Kochhar S , Garg P , Pandey AK , Dalal UR , Handa U . Screening Methods (Clinical Breast Examination and Mammography) to Detect Breast Cancer in Women Aged 40–49 years. J Midlife Health. 2017; 8(1): 2–10. 28458473 10.4103/jmh.JMH_26_16 PMC5367219

[9] Nelson HD , Tyne K , Naik A , Bougatsos C , Chan BK , Humphrey L , et al U.S. Preventive Services Task Force. Screening for Breast Cancer: An Update for the U.S. Preventive Services Task Force. Ann Intern Med. 2009; 151: 727–37. 19920273 10.1059/0003-4819-151-10-200911170-00009 PMC2972726

[10] Seely JM , Alhassan T . Screening for Breast Cancer in 2018-What Should We Be Doing Today? Curr Oncol. 2018; 25(Suppl 1): S115–S124. 29910654 10.3747/co.25.3770 PMC6001765

[11] Yip CH , Taib NA , Mohamed I . Epidemiology of Breast Cancer in Malaysia. Asian Pac J Cancer Prev. 2006 Jul; 7(3): 369–74. 17059323

[12] Li J , Shao Z . Mammography Screening in Less Developed Countries. Springerplus. 2015; 4: 615 10.1186/s40064-015-1394-8. 26543750 PMC4627993

[13] Rivera-Franco MM , Leon-Rodriguez E . Delays in Breast Cancer Detection and Treatment in Developing Countries. Breast Cancer (Auckl). 2018; 12: 1178223417752677 10.1177/1178223417752677. 29434475 PMC5802601

[14] Arslan AA , Formenti SC . Mammography in developing countries: the risks associated with globalizing the experiences of the Western world. Nature Clinical Practice Oncology. 2009; 6(3): 136–7. 19048009 10.1038/ncponc1282

[15] CRUK. Breast Module of the Cancer Awareness Measure (Breast- CAM) Toolkit. Cancer Research UK; 2011 Available at: https:// www.cancerresearchuk.org/sites/default/files/health_professional_breast_ca m_toolkit_09.02.11.pdf. Accessed October 24, 2018.

[16] Caviness LL , Cunningham DB , Gibson L . National Survey on Drug Use and Health. Research Triangle Park, NC: RTI International, 2006.

[17] Given , Lisa M . The SAGE Encyclopedia of ualitative Research Methods. Thousand Oaks, CA: SAGE Publications, Inc, 2008 10.4135/9781412963909.

[18] Adel A . Rapport Building in Student Group Work. J Pragmat. 2011; 43(12): 2932–47. 10.1016/j.pragma.2011.05.007.

[19] Bartkowiak J . NLP in qualitative research. [Opinion]. Int J Market Res. 2012; 54(4), 451–3. 10.2501/ijmr-54-4-451-453.

[20] WCRF. 2007 Food, Nutrition, Physical Activity, and the Prevention of Cancer: A Global Perspective. Washington D.C.; American Institute of Cancer Research, 2007.

[21] McDonald JA , Goyal A , Terry MB . Alcohol Intake and Breast Cancer Risk: Weighing the Overall Evidence. Curr Breast Cancer Rep. 2013; 5(3): 10.1007/s12609-013-0114-z. PMC3832299 24265860

[22] Seitz HK , Pelucchi C , Bagnardi V , La Vecchia C . Epidemiology and Pathophysiology of Alcohol and Breast Cancer: Update 2012. Alcohol Alcohol. 2012; 47(3): 204–12. 22459019 10.1093/alcalc/ags011

[23] Dumitrescu RG , Shields PG . The Etiology of Alcohol-induced Breast Cancer. Alcohol. 2005; 35(3): 213–25. 16054983 10.1016/j.alcohol.2005.04.005

[24] Oyesanmi O , Snyder D , Sullivan N , Reston J , Treadwell J , Schoelles KM . Alcohol Consumption and Cancer Risk: Understanding Possible Causal Mechanisms for Breast and Colorectal Cancers. Evid Rep Technol Assess. 2010; 197: 1–151. PMC4781486 23126574

[25] Ng J , Shuryak I . Minimizing Second Cancer Risk Following Radiotherapy: Current Perspectives. Cancer Manag Res. 2014; 7: 1–11. 10.2147/CMAR.S47220. 25565886 PMC4274043

[26] Travis LB , Hill D , Dores GM , Gospodarowicz M , van Leeuwen FE , Holowaty E , et al Cumulative Absolute Breast Cancer Risk for Young Women Treated for Hodgkin Lymphoma. J Natl Cancer Inst. 2005; 97: 1428–37. 16204692 10.1093/jnci/dji290

[27] Joosten A , Bochud F , Moeckli R . A Critical Evaluation of Secondary Cancer Risk Models Applied to Monte Carlo Dose Distributions of 2-dimensional, 3-dimensional Conformal and Hybrid Intensity- modulated Radiation Therapy for Breast Cancer. Phys Med Biol. 2014; 59(16): 4697–722. 25082795 10.1088/0031-9155/59/16/4697

[28] ACS. Breast Cancer Information Sheets; 2016 Available at: http:// www.cancer.org/acs/groups/cid/documents/webcontent/003090-pdf. Accessed October. 24, 2018.

[29] Parhizkar S , Nazari MR , Salleh MH . Breaking Taboo in Media Concerning Breast and Cervical Cancer. Asian J Soc Sci Humanit. 2012; 1(4): 49–55.

[30] Mendoza-Dreisbach S , Dreisbach JL . Female Breast Cancer as Taboo: Cultural Factors and Awareness Amongst Patients and Their Families in the Philippines. Geografia Malays J Soc Space. 2018; 14(4). 10.17576/GE0-2018-1404-16.

[31] Brunswick N , Wardle J , Jarvis MJ . Public Awareness of Warning Signs for Cancer in Britain. Cancer Causes Control. 2001; 12: 33–7. 11227923 10.1023/a:1008975416756

[32] Lundqvist A , Andersson E , Ahlberg I , Nilbert M , Gerdtham U . Socioeconomic Inequalities in Breast Cancer Incidence and Mortality in Europe-A Systematic Review and Meta-analysis. Eur J Public Health. 2016; 26(5): 804–13. 27221607 10.1093/eurpub/ckw070 PMC5054273

[33] Khakbazan Z , Taghipour A , Latifnejad Roudsari R , Mohammadi E . Help Seeking Behaviour of Women with Self-Discovered Breast Cancer Symptoms: A Meta-Ethnographic Synthesis of Patient Delay. PLoS One. 2014; 9(12): 110–262. 10.1371/journal.pone.0110262 PMC4254513 25470732

[34] Liu LY , Wang YJ , Wang F , Yu LX , Xiang YJ , Zhou F , et al Factors Associated With Insufficient Awareness of Breast Cancer Among Women in Northern and Eastern China: A Case-control Study. BMJ Open. 2018; 8(2): e018523 10.1136/bmjopen-2017-018523. PMC5855304 29463589

[35] Suls JM , Goodkin F . 1994 Medical Gossip and Rumor: Their Role in the Lay Referral System. Good Gossip 1994; 169–79.

[36] de Groot JE , Broeders MJ , Grimbergen CA , den Heeten GJ . Pain- preventing Strategies in Mammography: An Observational Study of Simultaneously Recorded Pain and Breast Mechanics Throughout the Entire Breast Compression Cycle. BMC Womens Health. 2015; 15: 26. 25783657 10.1186/s12905-015-0185-2 PMC4369109

[37] Chui PL , Abdullah KL , Wong LP , Taib NA . Prayer-for-health and Complementary Alternative Medicine Use Among Malaysian Breast Cancer Patients During Chemotherapy. BMC Complement Altern Med. 2014 Dec; 14(1): 425. 25358688 10.1186/1472-6882-14-425 PMC4230750

[38] Matsuno RK , Pagano IS , Maskarinec G , Issell BF , Gotay CC . Complementary and Alternative Medicine Use and Breast Cancer Prognosis: A Pooled Analysis of Four Population-based Studies of Breast Cancer Survivors. J Womens Health (Larchmt). 2012; 21: 1252–8. 23075455 10.1089/jwh.2012.3698 PMC3810617

[39] Norsa’adah B , Rahmah MA , Rampal KG , Knight A . Understanding Barriers to Malaysian Women with Breast Cancer Seeking Help. Asian Pac J Cancer Prev. 2012; 13: 3723–30. 23098462 10.7314/apjcp.2012.13.8.3723

[40] Harrington SE , Smith TJ . The Role of Chemotherapy at the End of Life: “When is Enough, Enough?”. JAMA. 2008; 299(22): 266778. 10.1001/jama.299.22.2667 PMC3099412 18544726

[41] Agide FD , Sadeghi R , Garmaroudi G , Tigabu BM . A Systematic Review of Health Promotion Interventions to Increase Breast Cancer Screening Uptake: from the Last 12 Years. Eur J Public Health. 2018; 28(6): 1149–55. 29351597 10.1093/eurpub/ckx231 PMC6241206

[42] Thune I , Brenn T , Lund E , Gaard M . Physical Activity and the Risk of Breast Cancer. N Engl J Med. 1997; 336: 1269–75. 9113929 10.1056/NEJM199705013361801

[43] Graf C , Wessely N . Physical Activity in the Prevention and Therapy of Breast Cancer. Breast Care (Basel). 2010; 5(6): 389–94. 21494404 10.1159/000322650 PMC3076351

[44] Bottorff JL , McKeown BS , Carey J , Haines R , Okoli C , Johnson KC , et al Women’s Responses to Smoking and Breast Cancer Risk Information. Health Educ Res. 2010; 25: 669–77. 10.1093/her/cyp067 PMC2905920 20080807

[45] Cummings KM , Brown A , O’Connor R . The Cigarette Controversy. Cancer Epidemiol Biomarkers Prev. 2007; 16(6): 1070–6. 10.1158/1055-9965.epi-06-0912. 17548665

